# Diagnosis and surgical approach in treating glomus tumor distal phalanx left middle finger: A case report

**DOI:** 10.1016/j.ijscr.2023.108426

**Published:** 2023-06-18

**Authors:** Moh. Asri Abidin, Muh. Ihsan Kitta, Ira Nong, Nur Rahmansyah, Muhammad Phetrus Johan

**Affiliations:** a Medical Faculty of Muhammadiyah University, Makassar, Indonesia; b Department of Orthopaedic and Traumatology, Faculty of Medicine, Hasanuddin University/Dr. Wahidin Sudirohusodo General Hospital, Makassar, Indonesia; c Medical Faculty of Bosowa University, Makassar, Indonesia

**Keywords:** Glomus tumor, Subungual glomus, Rare mesenchymal neoplasm, Transungual approach, Case report

## Abstract

**Introduction and importance:**

Glomus tumors are rare mesenchymal neoplasms. These tumors originate from the glomus bodies and are commonly found in the fingertips, especially in the subungual location. The cause of this tumor is unknown. Clinically, the symptoms are non-specific and often not identified on physical examination and radiologically accompanied by rare cases, making it difficult to diagnose a glomus tumor.

**Case presentation:**

Current report present a case of pain at the tip of the middle finger of the left hand in a woman for six years and worsening in the last two years. The patient has visited several doctors with analgesic therapy, but the complaints have not improved. A bluish nail was found on physical examination, and a clinical study with the Love's pin test and the Hildreth test had positive results. Radiographic examination showed destruction with cortical thinning of the medial aspect of the distal phalanx of the left middle finger, and MRI showed a lesion with an erosion of the distal middle finger. In this case, complete surgical excision and biopsy were performed using a transungual surgical approach. The sample was sent for microscopic examination, showing a glomus tumor.

**Clinical discussion:**

Cases with clinical symptoms of intense paroxysmal pain, exquisite point tenderness, and sensitivity to cold allow a clinical diagnosis in 90 % of cases. On clinical examinations such as Love's pin test, Hildreth's test, cold sensitivity test, and trans-illumination test with positive results and confirmed by MRI or ultrasound, the diagnosis of glomus tumor can be established.

**Conclusion:**

This case shows a glomus tumor in the distal phalanges of the middle finger of the left hand—diagnosis enforcement through detailed history taking and physical examination, confirmed by MRI and microscopic examination. Complete surgical excision is an effective treatment. In this case, using a transungual surgical approach based on preoperative MRI, the subungual lesion was found to provide the best exposure.

## Highlights

• Glomus tumor is a rare case, presenting with a triad of classic clinical symptoms that characterize glomus tumors, namely severe pain, pinpoint pain, and cold sensitivity
• Love's pin test, Hildreth's test, and cold sensitivity test are clinically specific tests used to confirm or deny symptoms in establishing a clinical diagnosis.
• The transungual surgical approach, in this case, allows the surgeon to complete the excision of the lesion with a subungual location

## 1 Introduction and importance

Glomus tumors are rare mesenchymal neoplasms. These tumors originate from the glomus bodies [[1], [2], [3], [4], [5]]. The glomus bodies are a highly specialized neuromyoarterial structure containing a delicate network of arteriovenous anastomoses that control blood pressure and temperature by regulating flow in the skin microvasculature [2,[5], [6], [7], [8], [9], [10], [11], [12]].

Epidemiologically, these tumors are common in young adults, both women, and men, but can also occur in a wider age range, namely 30–50 years. The frequency of tumors occurring in the extremities is only 1.5 % of all neoplasms in the extremities, but specifically for the subungual location, there are approximately 75 % of cases in women. Cases are more common in men when the tumor is located other than the subungual location [1,3]. Glomus tumors are often found in the fingertips. Although glomus tumors can occur anywhere on the body, up to 75 % occur in the hands, and approximately 65 % are on the fingertips, mainly in the subungual location. In addition, only 10 % or fewer glomus tumors are found in the volar pulp of the finger [1,2,4,13,14].

The trigger factor or the cause of this tumor is unknown. Chromosomal studies have identified at least three genes related to the tumor located on the long arm of chromosome 11. A familial variant of glomangioma has been linked to chromosome 1p21–22 and involves truncating mutations in the glomulin gene, which encodes a 68-kDa protein with unknown function [1,5,15]. This gene is autosomal dominant and is a very rare gene that is inherited. Reactive hypertrophy following trauma has been suggested to be caused by a structural weakening in the glomus body by some writers [5]. Although most are benign lesions with a tumor size of no more than 1 cm and solitary, these tumors can also be malignant [1].

Clinically, it is challenging to diagnose glomus tumors. Some patients sometimes experience a long duration of pain due to delays in diagnosis. Non-specific symptoms and the usual physical examination make an inaccurate diagnosis, so inappropriate treatment often occurs [1,4]. However, some patients are checked early because of the annoying pain. Still, the size of the tumor is the main obstacle because it is often not identified by physical and radiological examination [1].

Cases of glomus tumor, which is a rare case, patients come with complaints of pain that are felt only at the fingertips. Physical examination and radiographs are normal; in some cases complicated to identify early. Therefore, the discussion of glomus tumors is fascinating, especially since they can be identified from physical examination by performing special tests that lead to glomus tumors.

In this case report, we present a 35-year-old woman's complaints of pain in the middle finger of her left hand, which has been felt since six years ago and has worsened in the past two years. The study has been reported in accordance with SCARE 2020 standards [16].

## 2 Case presentation

We report a case of a 35-year-old woman who came with complaints of pain in the tip of the middle finger of her left hand for the past six years, and has worsened in the past two years. The patient has often been treated since she first felt the pain and only consumed pain killer, but the complaint did not change. The pain was supposed to increase when the fingertip was touched and pressed. There was no history of previous trauma.

**Fig. 1:**
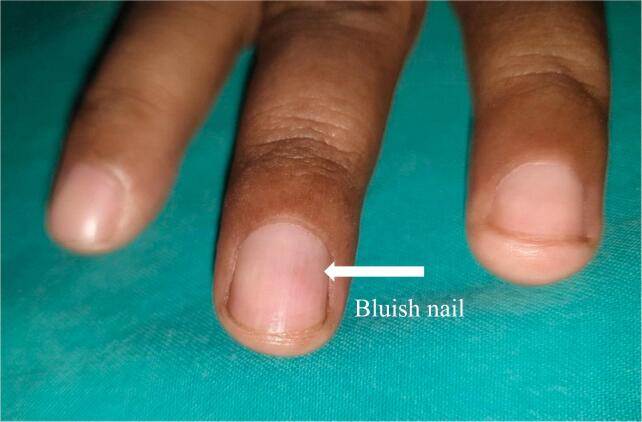
Bluish discoloration of the nail plate of the left middle finger because of a subungual glomus tumor.

On physical examination, generally, no abnormalities were found. On the assessment of the localized status on the middle finger of the left hand, there was a bluish nail (Fig. 1), there was no mass, swelling, redness, deformity, and the appearance of intact skin with the same color as the surrounding skin, clinical examination with the Love's pin test and the Hildreth test with positive results. Range of motion DIP, PIP within normal limits, and capillary refill time of fewer than 2 s.

**Fig. 2:**
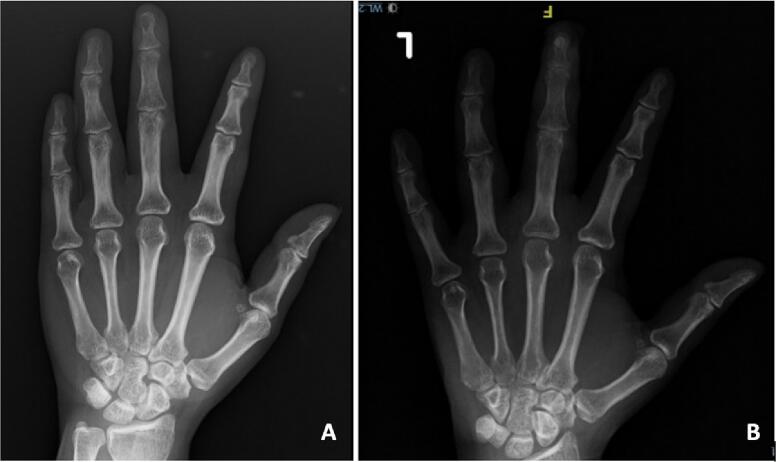
The anteroposterior radiograph shows bone destruction and cortical thinning in the distal phalanx of the left middle finger (A); the postoperative X-ray shows a synthetic bone substitute (B).

On plain radiographic examination of the anteroposterior view, there was destruction with cortical thinning on the medial aspect of the distal phalanx of the left middle finger (Fig. 2A).

**Fig. 3:**
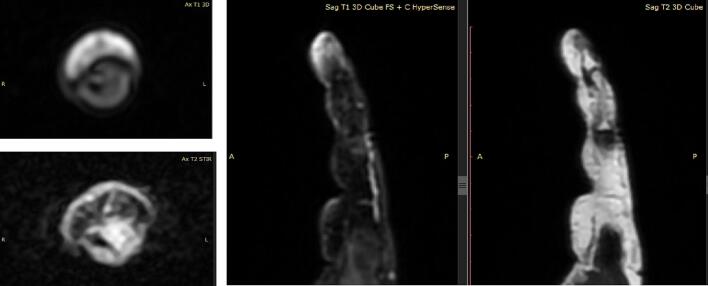
On MRI examination, the lesions with an erosion of the head of the distal phalanx of the middle finger.

On MRI examination, the lesion with size ± 0.43 × 0.90 × 0.56 cm in the distal phalanx of the left hand's middle finger with the head's erosion (Fig. 3).

**Fig. 4:**
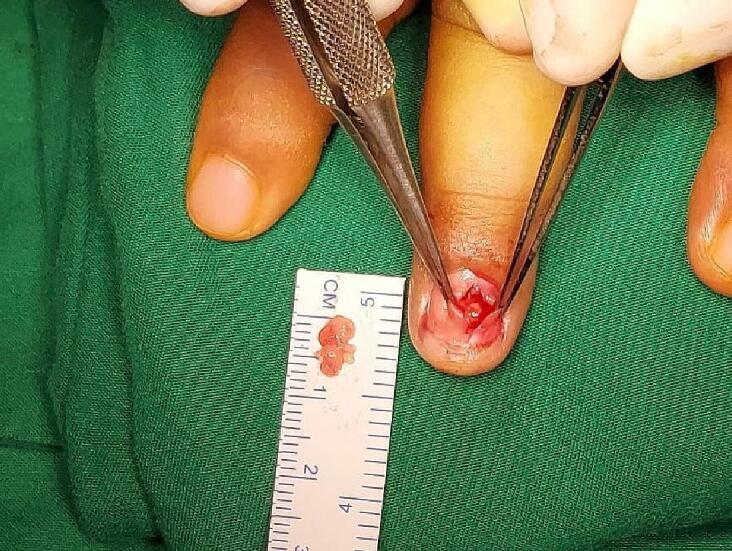
A transungual approach to the middle finger of the left hand obtained a mass with a chewy solid consistency in the subungual middle finger.

Based on history taking, physical examination, plain X-ray, and MRI, we decided to do an excisional biopsy. Surgery is performed by marking the specific location of the tumor. Local anesthesia was done with a digital nerve block and digital exsanguination and tourniquet for a relatively avascular operating field. Excision with a transungual surgical approach, by experienced oncologic orthopaedic surgeon assisted by experienced hand and microsurgery surgeon, based on the tumor's location on the MRI image to facilitate exposure of the tumor site. A periosteal elevator is inserted between the nail and the nail bed. The nail is removed, being careful not to damage the nail bed. Then the nail is removed from the bed and nail fold, exposing the nail bed, and a tumor is visible under the nail bed. The nail bed is incised longitudinally, and the tumor is carefully removed and completely removed. A synthetic bone substitute was placed to fill the defect in the distal phalanx. The primary suture was made using absorbable Vicryl 7-0 suture, and the nail plate was repositioned after being covered with nylon 4-0. Wound dressing is done using antibiotic cream and gauze. The excised tissue is then sent to the anatomic pathology department for microscopic histopathological examination (Fig. 4).

**Fig. 5:**
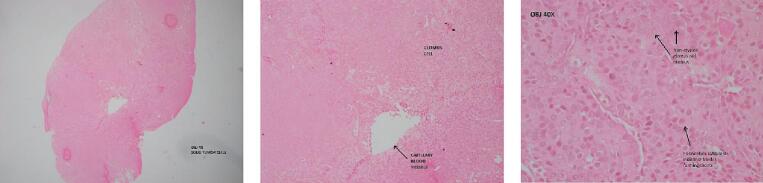
On microscopic examination, the specimen shows a tumor mass that consists of a proliferation of glomus cells.

On microscopic examination, the specimen shows a tumor mass that consists of a proliferation of glomus cells. There are capillary vessels that are lined by endothelial cells surrounding the tumor mass. Glomus cell has round to oval nuclei, no atypia, and inconspicuous nucleoli, with eosinophilic cytoplasm with a sheet-like pattern. Between tumor mass, spindle cell also suggests smooth muscle origin (Fig. 5). Based on microscopic examination, it can be concluded that the specimen is a glomus tumor.

Postoperative X-ray showing a synthetic bone substitute filling the defect in the distal phalanx of the left middle finger (Fig. 2B).

## 3 Clinical discussion

Glomus tumors are benign hamartomas from the normal glomus apparatus located in the subcutaneous tissue. The normal glomus body is a contractile neuromyoarterial receptor that controls blood pressure and temperature by regulating flow in the cutaneous microvasculature. The glomus bodies are highly concentrated in the fingertips, especially under the nails, so the tumor is usually located in the subungual area [[5], [6], [7]].

According to their clinical presentation, glomus tumors are categorized into solitary and multiple types. Solitary glomus tumors are painful and occur mainly in the distal extremities; some glomus tumors are usually painless and can develop in any part of the body [17].

This glomus tumor consists of 3 components: glomus cells, blood vessels, and smooth muscle cells. Based on the proportion of these three components, glomus tumors are divided into three subcategories: glomus solid tumors, glomangiomas, or glomangiomyomas. Glomus solid tumors were the most frequent subtype (75 %), followed by variant glomangiomas (20 %) and glomangiomyomas (5 %). This subcategory is based on the dominant component. The vascular and smooth muscle components are very few in solid glomus tumors. In glomangiomas, the vascular feature is more prominent, and in glomangiomyomas, both vascular and smooth muscle is equally notable. In this case, based on the histopathological results, it was categorized as a glomus solid tumor [1].

These glomus tumors are often small, located deep in the fingertips, and are usually not palpable, making them difficult to diagnose. A common characteristic in most cases is a long duration of symptoms before proper diagnosis and treatment are established [4]. The delay in diagnosis, in this case, was six years. During this period, the patient is in pain due to an error in the initial diagnosis. The patients visit many doctors and other health professionals without a definite diagnosis or treatment plan. Diagnostic delays can occur for several reasons. For example, severe pain may be mistaken for other causes such as neuromuscular pain or chronic regional pain syndrome (CRPS), neuroma, gouty arthritis, haemangioma, etc. [2,4,5,10] A relatively long duration from symptom to diagnosis has been reported in the literature. Yilmaz et al. noted a median delay of seven years and four months due to misdiagnosis in their study [18].

Most physicians do not understand the triad of classic clinical symptoms that characterize glomus tumors, namely intense paroxysmal pain, exquisite point tenderness, and sensitivity to cold [1,3,[5], [6], [7], [8],[13], [14], [15],[19], [20], [21], [22]]. Pain is a common complaint accompanied by two other symptoms found in varying proportions of patients. This triad is considered to allow clinical diagnosis in 90 % of cases [2,4].

On local examination, the tumor is often not visible and not palpable. In rare cases, one may notice a visual space occupying the lesion under the nail plate, a purplish pink discoloration under the nail, or some visible deformation of the nail plate itself. Specific clinical tests are usually used to confirm or deny the symptoms in establishing a clinical diagnosis, such as the Love's pin test, Hildreth's test, cold sensitivity test, and trans-illumination test [2,21].

The Love's pin test is used to confirm exquisite point tenderness. A pinhead is used to apply gentle local pressure to the painful area as indicated by the patient subjectively, which usually increases the pain scale. In the Hildreth test, transient ischemia is produced by applying a tourniquet to the upper arm. In the presence of ischemia, when The Love's pin test was carried out, there was no pain like the previous examination. After removing the tourniquet, there is a quick return of the bothering pain. A cold intolerance test is performed by applying an ice cube to the affected area. The trans-illumination is obtained by shining light through the finger pad in a dark room. The cold sensitivity test was reported to have a sensitivity, specificity, and diagnostic accuracy of 100 %. The Love test has a sensitivity of 100 % and a diagnostic accuracy of 78 %, while the Hildreth test has a sensitivity of 71.4 %, a specificity of 100 %, and a diagnostic accuracy of 78 % [2,5,[10], [11], [12], [13], [14], [15],^18^].

**Fig. 6:**
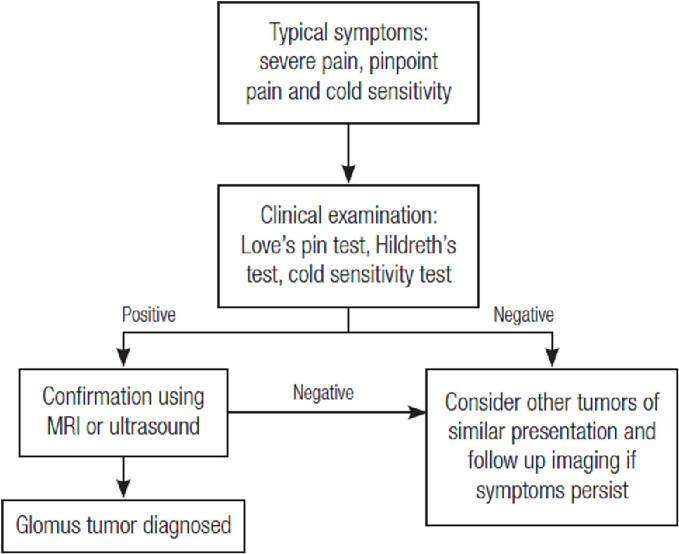
Diagnostic algorithm for glomus tumor.

Some literature states that in confirming the diagnosis of glomus tumors, in addition to special examinations that can be done to localize the lesion and diagnosis, ultrasound and MRI investigations can also be carried out to assist in the diagnosis and planning of surgical management [1,10,18]. An essential guide on the steps for diagnosing a glomus tumor is demonstrated through a simple diagnosis algorithm (Fig. 6) [10].

Preoperative MRI, a transungual approach with ideal access to the subungual tumor, and a magnification loupe may all be factors that facilitate complete tumor excision and hence no recurrence. Several published studies have also reported no recurrence in operated patients [2].

Tumor microscopy and histology glomus comprise a proliferation of neoplastic cells forming a solid pattern. Cell morphology is spherical with a small size and tends to be uniform with a round nucleus located in the center; cytoplasm is amphophilic to eosinophilic. A well-defined basal lamina surrounds each cell. Occasional oncocytic and epithelioid changes may also be seen. Solid glomus tumor nests surrounding the capillaries. The area around the stroma shows hyalinization or myxoid changes. This picture is following found in the case [1].

Complete surgical excision of the tumor is the only effective treatment. Incomplete excision is considered the main cause of recurrence. Preoperative MR imaging can be useful to delineate the tumor better and for proper excision, reducing the chance of recurrence. The recurrence rate varies from 12 % to 33 %. It is generally thought that symptoms reappearing in the days to weeks following surgery may indicate inadequate excision; conversely, symptoms, when they appear 2 to 3 years postoperatively, may indicate multiple tumors [4,5,8,11,[23], [24], [25]].

Surgical approaches to glomus tumors via transungual and lateral subperiosteal approaches have been widely reported [14,19]. The transungual approach to excision of the glomus tumor is usually recommended [13,19] because it gives the best exposure if the lesion is entirely subungual. While this approach offers a good field of view, it can produce unsatisfactory cosmetic results if the nail bed is severely damaged when removing the tumor or if nail bed sutures are made without treatment, leading to postoperative nail deformity [14,17,19]. In this case, a transungual approach was used with the subungual lesion. Several authors have used a lateral approach, i.e., lateral subperiosteal and later-ungual. The main disadvantage reported with the lateral approach is that it is difficult to expose the nail bed in subungual lesions, especially in the case of tiny tumors [4,13].

## 4 Conclusion

These glomus tumors are often small, located deep in the fingertips, and are usually not palpable, making them difficult to diagnose. General practitioners and family physicians may be the first health workers to see these patients. Performing a meticulous history taking and physical examination is very important in establishing the diagnosis of glomus tumor. Complete excision surgery using a transungual surgical approach for subungual lesions provides good exposure, making it easier for the surgeon to perform a complete excision.

## Provenance and peer review

Not commissioned, externally peer-reviewed.

## Consent

Written informed consent was obtained from the patient for publication of this case report and accompanying images. A copy of the written consent is available for review by the Editor-in-Chief of this journal on request.

## Ethical approval

The study is exempt from ethical approval in our institution. Our institutional review board does not provide an ethical approval in the form of case report.

## Funding

None.

## Guarantor

Muhammad Phetrus Johan.

## CRediT authorship contribution statement

Moh. Asri Abidin (AB): Conceptualization, Writing the paper, and English checking
Muh. Ihsan Kitta (MI): Editing, English checking, and manuscript reviewing.
Ira Nong (IO): Assistant of a surgeon, editing, English checking, and manuscript reviewing
Nur Rahmansyah (NS): Editing, English checking, and manuscript reviewing.
Muhammad Phetrus Johan (MPJ): Surgeon, conceptualization, and writing the paper.

All authors discussed the results and contributed to the final manuscript.

## Conflicts of interest

The authors declare that there are no conflicts of interest regarding the publication of this article.
